# Automatic generation of structural geometric digital twins from point clouds

**DOI:** 10.1038/s41598-022-26307-7

**Published:** 2022-12-24

**Authors:** Kaveh Mirzaei, Mehrdad Arashpour, Ehsan Asadi, Hossein Masoumi, Heng Li

**Affiliations:** 1 Department of Civil Engineering, Monash University, Melbourne, Australia; 2 School of Engineering, RMIT University, Melbourne, Australia; 3 Department of Building and Real Estate, Hong Kong Polytechnic University, Hung Hom, Hong Kong

**Keywords:** Engineering, Optics and photonics

## Abstract

A geometric digital twin (gDT) model capable of leveraging acquired 3D geometric data plays a vital role in digitizing the process of structural health monitoring. This study presents a framework for generating and updating digital twins of existing buildings by inferring semantic information from as-is point clouds (gDT’s data) acquired regularly from laser scanners (gDT’s connection). The information is stored in updatable Building Information Models (BIMs) as gDT’s virtual model, and dimensional outputs are extracted for structural health monitoring (gDT’s service) of different structural members and shapes (gDT’s physical part). First, geometric information, including position and section shape, is obtained from the acquired point cloud using domain-specific contextual knowledge and supervised classification. Then, structural members’ function and section family type is inferred from geometric information. Finally, a BIM is automatically generated or updated as the virtual model of an existing facility and incorporated within the gDT for structural health monitoring. Experiments on real-world construction data are performed to illustrate the efficiency and precision of the proposed model for creating as-is gDT of building structural members.

## Introduction

The structural health of building members affects the lives and safety of the public^1^. Monitoring structural health consists of on-site observations, condition evaluation, data management, decision-making, planning, and executing the required repairs^2^. Conventional monitoring methods rely on visual inspection and manual measurements of structural members, which are tedious and error-prone^3^. Also, the effectiveness of procedures depends on the skill and self-discipline of the responsible personnel^4^. Considering a large number of aging buildings and infrastructure projects^5^, an automated framework for proactive and accurate structural assessment and health monitoring is of the utmost importance in the Architecture, Engineering, and Construction (AEC) industry^6^.

Digital Twins (DTs) have gained a plethora of attention due to improvements in data acquiring technologies, data processing and simulation capabilities, and accessibility of computing infrastructure^7,8^. DT^9–11^ is a comprehensive tool for collecting and simulating information with a feedback loop to ensure the coordination of the physical spaces and the digital model of cyberspace during the entire life cycle of a project for reasoning and decision-making^8^. The DT concept has been extensively developed in the manufacturing industry in different life cycle stages such as design stage^8^ and configuration stage^12,13^ and also for a variety of applications including but not limited to parallel controlling of smart workshop^14^, designing of board-type furniture production line^15^, and designing of automated flow-shop manufacturing system^16^. In the construction industry, DT has substantially contributed to improvements in applications such as construction quality monitoring and management^17^, defect detection in construction projects^18^, and construction asset monitoring^19,20^. However, the lack of efficient and accurate algorithms, software, and clearly defined modeling procedures for building structural members hinders DT’s development for structural health monitoring purposes^21^.

A DT consists of five main parts: (1) physical part; (2) virtual model; (3) connection, which is the device or technique used for obtaining data for integrating virtual and physical spaces^14^; (4) data that is obtained from the physical part; and (5) service, which is the target application of DT^22^. The foremost step and cornerstone of creating an efficient and accurate DT for structural health monitoring purposes is to generate a virtual geometric representation of the as-is asset^23^ in a parametric and updatable platform^21^, also known as a geometric Digital Twin (gDT)^23,24^. Building Information Model (BIM) has been proven to allow incorporating semantically rich information for a gDT modeling approach throughout the life cycle of a building to help various applications, including building structural health monitoring and maintenance^25^. To adopt BIM as the gDT’s virtual model for structural health monitoring purposes, it should be accurate and contain updated information on building structural members. However, most buildings’ existing information on structural members is out-of-date^26^ and primarily stored as 2D drawings in hard copy and/or electronic Computer-Aided Design (CAD) formats^23^. The current approach for creating as-is BIM to be used as gDT’s virtual model is to manually obtain information from buildings^21,27^ and create/update BIM in modeling and simulation software such as Autodesk Revit, which is tedious, time-consuming, and lacks accuracy due to difficulties associated with accessing structural members in the majority of buildings^28^. Thus, there is a need to develop accurate and efficient methods capable of creating as-is BIM as gDT’s virtual model for building structural members.

Recently, the rapid development of surveying and non-contact sensing technologies has improved the accuracy and efficiency of generating gDTs’ virtual models of the existing facilities in the format of BIM^21^. Noteworthy examples of such devices (i.e., gDT connection devices) for acquiring accurate data are radio-frequency identification (RFID)^29^, 2D camera^30–32^, 3D camera^33^, and laser scanner^34–36^. Laser scanners have shown a high level of accuracy in capturing data of the physical part in point clouds format (i.e., gDT data part)^37^. Point clouds store 3D geometric information consisting of geometric or geodetic coordinates^38,39^. However, laser scanners cannot obtain semantic information (e.g., object class) required for generating BIMs as gDTs’ virtual models and data processing approaches are needed to obtain and infer semantic information.

Xue et al.^40^ demonstrated the use of 2D images for developing semantically rich as-built BIMs, in which they categorized semantic information into two main categories of (1) geometric information, such as member position, section shape, and dimensional tolerance, and (2) non-geometric information, such as member function (i.e., beam, column, etc.) and type (i.e., section family type). Geometric information can be obtained directly from point clouds for generating BIMs as gDT’s virtual models, non-geometric information should be interpreted and assessed from geometric information.

The first type of geometric information required for creating as-is BIM of structural members as gDT’s virtual model is position information consisting of (1) geometric definitions of structural members, such as columns being vertical members, and (2) spatial relationships between structural members, such as bracings positioned under beams^41^. Segmentation algorithms have been used to congregate points with similar geometric features of structural members^41^. Clustering algorithms^42^ were widely used to detect similar patterns within a point cloud, also known as clusters based on different features^43^, comprising spatial position^44^, points normal vector^45^, and density of points within point clouds^46^. While research efforts have focused on detecting structural members using position information^41^, relying solely on position information is error-prone^47^, sensitive to noise in point cloud datasets, and only labels point clusters as potential structural members.

Other research utilized machine learning methods, such as Convolutional Neural Networks (CNNs), to obtain section shape information from point clouds as the second type of geometric information required for creating an as-is virtual model of structural members for gDT creation. Machine learning methods have been deployed in various applications^48,49^, such as construction waste detection^50^. However, due to the irregularity, unorderdness, and unstructured nature of point cloud data, convolutional operations of CNN methods are incapable of being established^51^. Other works have deployed deep learning methods. The first group of these methods converts the point cloud into a structured grid^52^, which is memory-intensive and leads to losing much information^51^. The second group of methods, like PointNet^53^, directly applied deep learning to point clouds. PointNet acts as a local feature learner to generate a global point signature by aggregating individual point features. PointNet achieved state-of-the-art performance on a variety of benchmark datasets. Recent works have directly detected structural members^54^ and Mechanical, Electrical, and Plumbing (MEP) systems^55^ using machine learning-based methods. These methods required large numbers of pre-annotated real-world training datasets similar to their investigated case studies, which opposes a challenge for applying such techniques to other construction projects. Further research utilized slicing methods and image processing approaches to overcome such challenges for detecting section shapes within point cloud datasets^28,56^. The obtained geometric information was used to infer two ending points of the component centerline and the corresponding section shape information for automatically modeling MEP components in a BIM^28^. These research efforts achieved a high detection accuracy due to using matured image processing techniques and methods. However, the performance of such approaches is heavily impacted by the existence of large numbers of non-related objects in point clouds. Also, projecting 2D images to 3D space impacts the overall performance of such approaches.

Non-geometric information, inferred from point clouds, is also vital for creating the gDT’s virtual model of structural members. Recent works have inferred two ending points of the component centerline and the corresponding cross-section information for automatically modeling MEP components in a BIM^28^. While applicable for MEP components, the viability of this method for inferring non-geometric information from point clouds and modeling structural members in a BIM remains unproven.

Considering the body of research studied above^35,41,42,56–63^, challenges in the automated creation of gDT’s virtual model from as-is point clouds for building structural health monitoring can be summarized in four categories: (1) manual processing steps for removing noise and occlusions from point cloud data and manually classifying and detecting structural members, which is not efficient and practical for creating a gDT’s virtual model; (2) heavy reliance of previous research on accurate local features in point clouds may not be reliable for real-world point cloud datasets with high levels of noise and occlusions; (3) limited applications of previous research to a specific type of building structure member or a structural section shape, which cannot be generalized to other building types and structural member section shapes; and (4) the need for adopting various technologies that leads to complex data processing seps^23^. Due to the importance of efficiency, accuracy, and generalizability for the automated creation of gDT’s virtual model for realizing the target services of gDT, there is a strong need for methods capable of automatically obtaining semantic information from as-is point clouds and transforming such information into BIMs of building structural members^40,64^.

Therefore, this study aims to develop an automated, accurate, and generalizable framework for creating gDT of building’ structural members including beams, columns, and bracing members (gDT’s physical part) by obtaining and inferring semantic information from point clouds (gDT’s data) acquired from laser scanners (gDT’s connection) and storing it in updatable BIMs (gDT’s virtual model) for structural health monitoring purposes (gDT’s service). To reach this aim, we use geometric definitions and spatial relationships between structural members backed up by inputs from standards and regulations for automated detection of potential structural members and filtering out noises and non-structural members. Then, PointNet trained by synthetically generated models is implemented for detecting the section shape of structural members, followed by a general approach for inferring non-geometric information from point clouds using contextual information of building structural members. Finally, an Application Programming Interface (API) in Revit is developed to automatically model the detected structural members in a BIM. Specifically, we aim to answer the following research questions: (Q1) How is geometric information required for generating gDT obtained automatically from raw point cloud data? (Q2) What non-geometric semantic information is inferable from point clouds? (Q3) How can semantic information obtained from point clouds be translated into BIMs as gDT’s virtual models? (Q4) How accurate and efficient can the proposed automated point cloud processing method generate the as-is gDTs’ virtual model of structural members?

**Figure 1:**
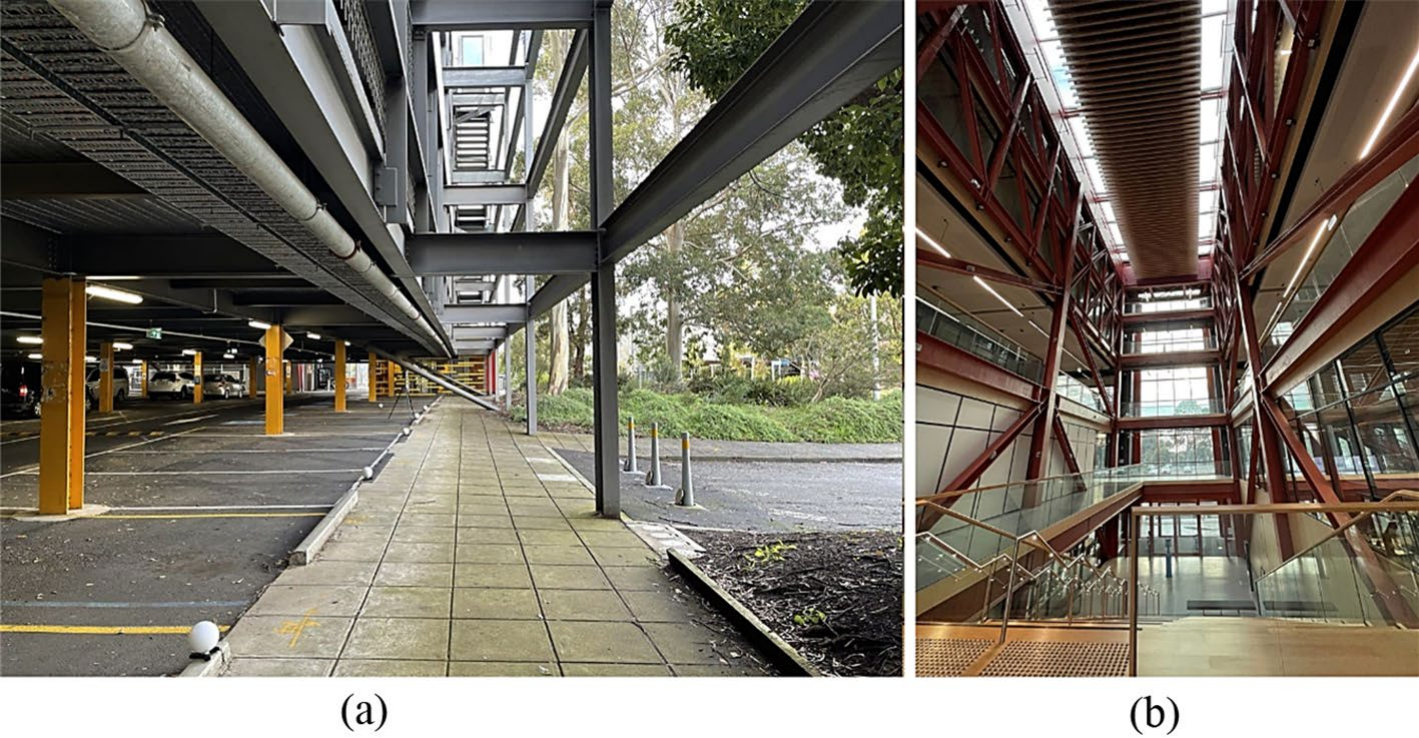
Image of the case study construction projects (**a**) multi-level carpark, and (**b**) Woodside building.

To answer Q1, we adopted a clustering method based on contextual hard-coded knowledge of structural members along with a shape detection method backboned by the PointNet trained by synthetic structural shapes to obtain position and shape information. To answer Q2, we defined a set of conditional rules for detecting each structural member’s function and section family type using its geometric information. We answered Q3 by creating a database of semantic information obtained from raw point clouds and connecting that to the database of Revit for creating BIMs. To answer Q4, we evaluated the performance of the proposed method by comparing the obtained dimensions from BIM to the ground truth dimensions and the time required for point cloud processing to the manual method of creating BIMs for two real-world case study construction projects (a) N1 Monash multi-level carpark (hereinafter referred to as multi-level carpark), and (b) Woodside Building for Technology and Design (hereinafter referred to as Woodside building), as shown in Fig. 1.

## Results

The results section is divided into obtaining geometric information from point clouds, inferring non-geometric information, and creating BIM steps. The assumptions regarding processing algorithms inputs are limited to the multi-level carpark case study; the Woodside building case study produces similar results.

### Obtaining geometric information from point clouds

**Figure 2:**
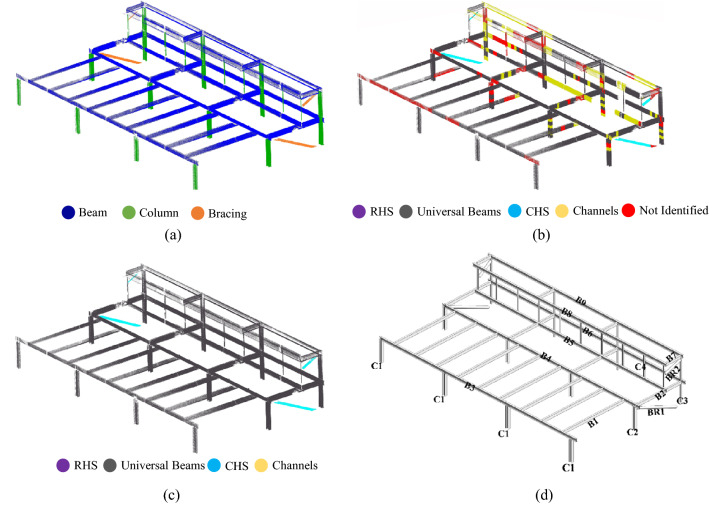
Applying the proposed method to the multi-level carpark case study: (**a**) Obtaining position information for detecting potential structural members. (**b**) Detection of section shapes. (**c**) Homogenization of structural shapes along the length of each member. (**d**) Automatically generated BIM model in Revit.

**Figure 3:**
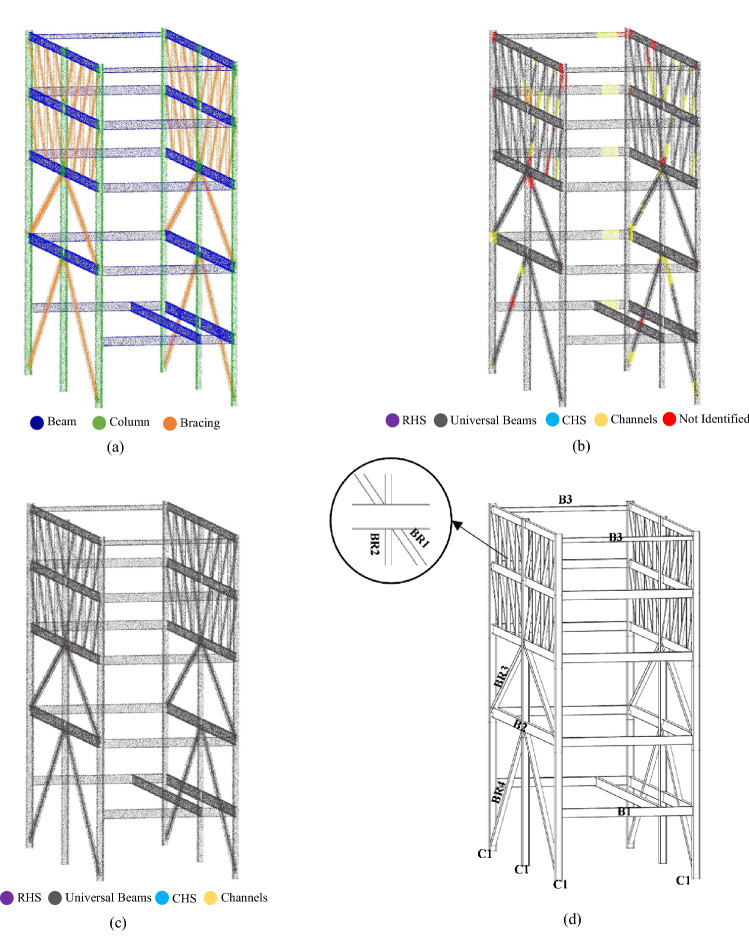
Applying the proposed method to the Woodside building case study: (**a**) Obtaining position information for detecting potential structural members. (**b**) Detection of section shapes. (**c**) Homogenization of structural shapes along the length of each member. (**d**) Automatically generated BIM model in Revit.

The first geometric information required for detecting structural members within a point cloud dataset is position information. For that, we adopted contextual hard-coded knowledge and geometric definition of beams, columns, and bracing members. In structural design concepts, beams are usually geometrically defined as horizontal members leading to the congestion of points associated with beams in horizontal slices within point clouds. Therefore, to detect points with the geometric definition of beams (i.e., potential beam points), we sliced the point clouds of case study buildings with horizontal planes. While a higher number of slicing planes results in more accurate results, it would be a computationally heavy process due to the high number of points that should be processed. Considering the proposed section shapes in AS/NZS 3679.1:2016^65–67^, the lowest difference between section heights is 20 mm. Thus, the number of horizontal slices should be identified to yield a slice thickness of less than 10 mm (considering both the top and bottom of beams) to obtain the correct section dimensions. Considering the ceiling height of roughly 4 m, we sliced the multi-level carpark case study point cloud with 400 horizontal planes. The average number of points within potential beam slices was 100,000 points and for slices with non-beam objects was 5000 points. Therefore, we extracted potential beam points using the statistical distribution of point numbers within slices. The outcome of this step is a point cloud of building beams. Thus, the MeanShift clustering method was utilized to cluster potential beam points based on the coordinates of each point to identify separate instances of beams. As for the point cloud investigated in this study, we used the MeanShift clustering function proposed in Scikit-learn with a quantile value of 0.4 and a sample number of 550^68^. The inputs of the MeanShift clustering algorithm were chosen based on the perpendicular distance of neighboring beam point clusters, which ranges from 3 to 6 m in typical buildings. Next, potential column points were geometrically segmented by estimating the point normal in the point cloud with a search radius of 0.7, meaning that points in the 0.7 m radius of each point were used for plane fitting and normal calculation^69^. Potential column points were detected by filtering out the points with an absolute normal vector value in the Z direction (*nZ)* lower than 0.02, as recommended in AS/NZS 5131:2016^70^ for the permissible inclination of columns. Finally, we identified potential bracing members by considering them as members beneath the bounding box of potential beam instances. Figures 2a and 3a illustrate the result of obtaining positional information for detecting potential structural members of multi-level carpark and Woodside building, respectively. Also, Table 1 includes the inputs and thresholds used for finding position information in both case study buildings.

**Table 1:** Inputs and thresholds used for finding position information from point clouds.

Algorithm input	Structural member	Multi-level carpark	Woodside building
No. of horizontal planes for slicing	Beams	400	400
MeanShift clustering quantile value	Beams	0.4	0.5
MeanShift clustering sample number	Beams	550	550
Normal vector search radius	Columns	0.7	0.8

The shape information of object instances found in the previous step is the second geometric information required for detecting structural members within a point cloud dataset. For that, we created a synthetic training dataset consisting of Circular Hollow Sections (CHSs), Universal Beams (UBs), Rectangular Hollow Section (RHSs), and Channels (Cs) for the training of the PointNet network used for shape detection. A total of 6000 synthetic 3D models evenly distributed between different categories were picked for training, and 2000 synthetic 3D models were used for validation. We then converted 3D models into point clouds by evenly distributing a predefined number of points on their surfaces. The predefined number of points for converting synthetic 3D models to point clouds is related to the density of points in the case study point cloud. This number should create a similar point density in the training dataset to the point density in the case study. Therefore, synthetic 3D models were automatically converted to point clouds with 15,000 points to create a point density in the training dataset samples similar to the point density generated by the laser scanner. Also, we applied a random rotation, noise, and occlusion to the training dataset to increase its similarity with the case study point cloud.

We trained the PointNet network with a dropout rate of 0.7, a learning rate of 0.001, and other hyperparameters, as suggested in its original paper^53^. The network training took 200 epochs using a batch size of 32 for converging the loss function, which took 20–21 h. Next, we sliced object instances found in the previous step to make them similar to the training dataset and removed the length of structural members as a feature to increase the performance of the shape detection network. The outcome of the shape detection network is a probability vector. Thus, we utilized the Probability Vector Length (PVL) parameter to evaluate the confidence of the shape detection network using Eq. (1). A high value of PVL demonstrates a high variation between the parameters of the probability vector, meaning that the shape detection had a higher level of confidence for detecting section shapes. The maximum value for the PVL is always equal to one, and the lowest value ($\sigma =0)$ is related to the population of the probability vector. Since four categories of section shapes for classification were defined, the minimum value for PVL, i.e., lowest network confidence for prediction, was 0.5. We set the threshold for the lowest acceptable PVL to be 0.75, and the members with PVL lower than this threshold were labeled as “Not identified”. Figures 2b and 3b depict the results obtained from the shape detection network for multi-level carpark and Woodside building point clouds, respectively.

1 $$PVL=\sqrt{n{\sigma }^{2}+\frac{1}{n}}.$$

### Inferring non-geometric information

Structural member function information is the first non-geometric information required to create building structural members’ virtual model. We manually labeled the “Not identified” point clouds using CloudCompare software. Then, considering the rarity of having multiple section shapes along structural members, we homogenized the section label along each instance by choosing the section label with the maximum number of points as the section label for the whole object instance, as shown in Figs. 2c and 3c for multi-level carpark and Woodside building point clouds, respectively. Next, we defined structural members as object instances that were geometrically labeled as structural members (beam, column, or bracing) with a structural section shape assigned to them and inferred their function by their geometric label.

The second non-geometric information required for creating a virtual model of structural members is section family type. To infer such information, we created a database consisting of structural member function (i.e., beam, column, bracing), section shape (i.e., UB, CHS, RHS, C), section width and height, member length, and section center points for the start and end of the structural member. Table 2 includes the obtained information from random point cloud examples within the dataset. This information was obtained by investigating the bounding box around each structural member. Moreover, UB cross-sections were divided into IPE and IPB members based on the proportion between section height and width. Also, section names were defined similarly to the default families in Revit to create a connection between the obtained information and BIM model generation software. Finally, x0, y0, and z0 are the coordinate of the center of the bounding box section at the starting location of the structural member, and x1, y1, and z1 are the coordinate of the center of the bounding box section at the end of the structural member.

**Table 2:** Information obtained from structural members point clouds.

	Section shape	Section name	Structure member function	x0	y0	z0	x1	y1	z1
1	IPE	310UB40	Beam	− 68.57	− 9.16	279.16	15.18	− 9.16	279.1
2	CHS	CHS273	Bracing	− 67.94	− 7.06	261.78	− 67.94	8.29	268.1
3	IPE	310UB40	Column	− 67.90	− 9.13	261.81	− 67.90	− 9.13	278.4
4	IPB	100UC15	Column	− 49.55	− 2.61	270.91	− 49.55	− 2.61	278.4
5	IPB	310UC98	Column	− 67.80	37.56	260.67	− 67.80	37.5	268.7
6	IPE	310UB40	Column	− 13.37	− 9.15	261.74	− 13.37	− 9.15	278.4

### Creating BIM

For creating a BIM of structural members from point cloud datasets to be used as gDT’s virtual model, we developed an API that connects the information stored in Table 2 to the database of Revit. The developed API reads each row of Table 2 for obtaining the required information such as section name, member length, member position, and member function for automatically creating object instances within the Revit, as depicted in Figs. 2d and 3d for multi-level carpark and Woodside building point clouds, respectively. The obtained BIM model is the as-is gDT virtual model of the case study buildings.

## Discussion

Two main approaches were proposed for obtaining position and section shape information from point clouds. We utilized structural members’ geometric definitions and spatial relationships to get position information. The advantage of the proposed slicing method for geometric segmentation of potential beam points is that it is irrespective of the noise level and non-structural points in the point cloud due to the immersive difference between the number of points in potential beam slices and non-beam slices. This advantage will increase the generality and repeatability of the proposed framework by eradicating the need for manual noise removal and point cloud clearing steps. Also, the proposed method demonstrated a satisfactory performance for detecting potential column points. One of the challenges of the proposed method is finding the optimized parameters for the MeanShift clustering method in cases where the distance between side-by-side beams is not constant, such as the multi-level carpark case study. Overall, using contextual hard-coded knowledge and geometric definition of beams, columns, and bracing members not only segments out potential points belonging to structural members but also improves the performance of other steps by reducing the computational burden for the following steps by decreasing the number of points and also, removing noises and non-structural members without geometric definitions of structural members.

**Figure 4:**
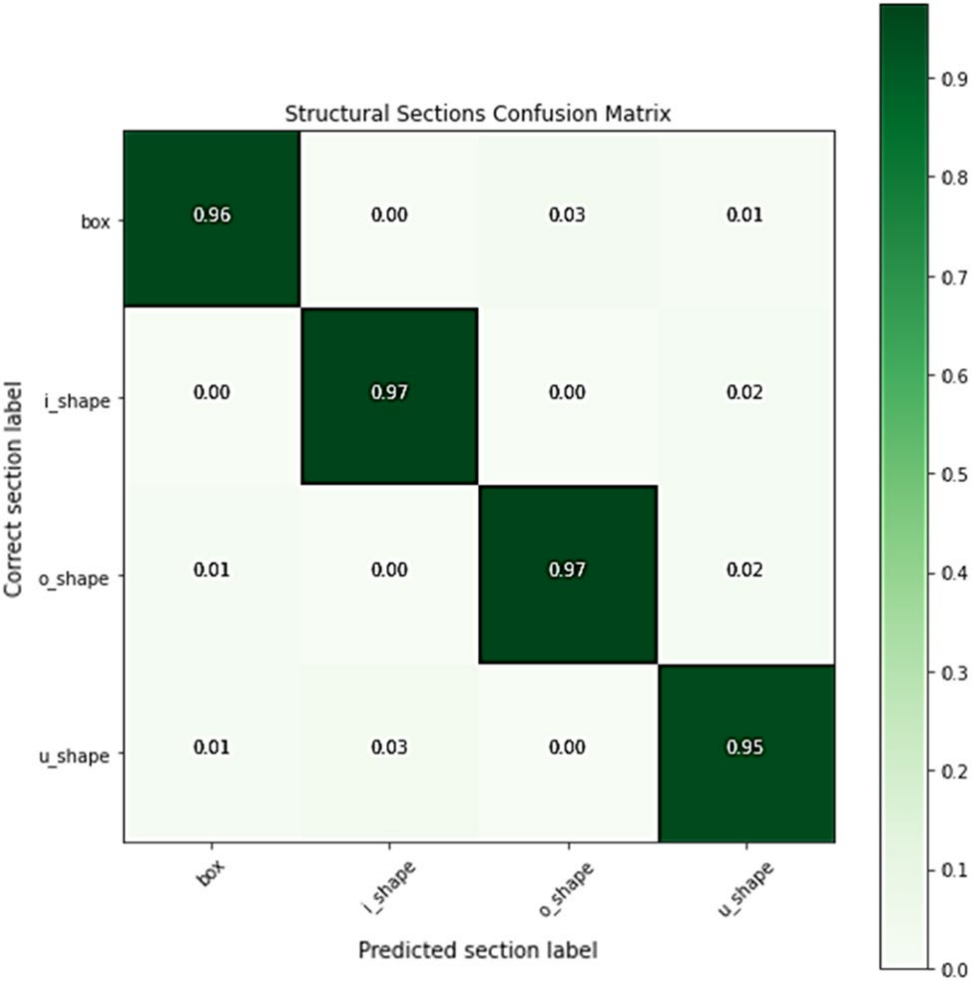
PointNet performance in the validation dataset.

**Figure 5:**
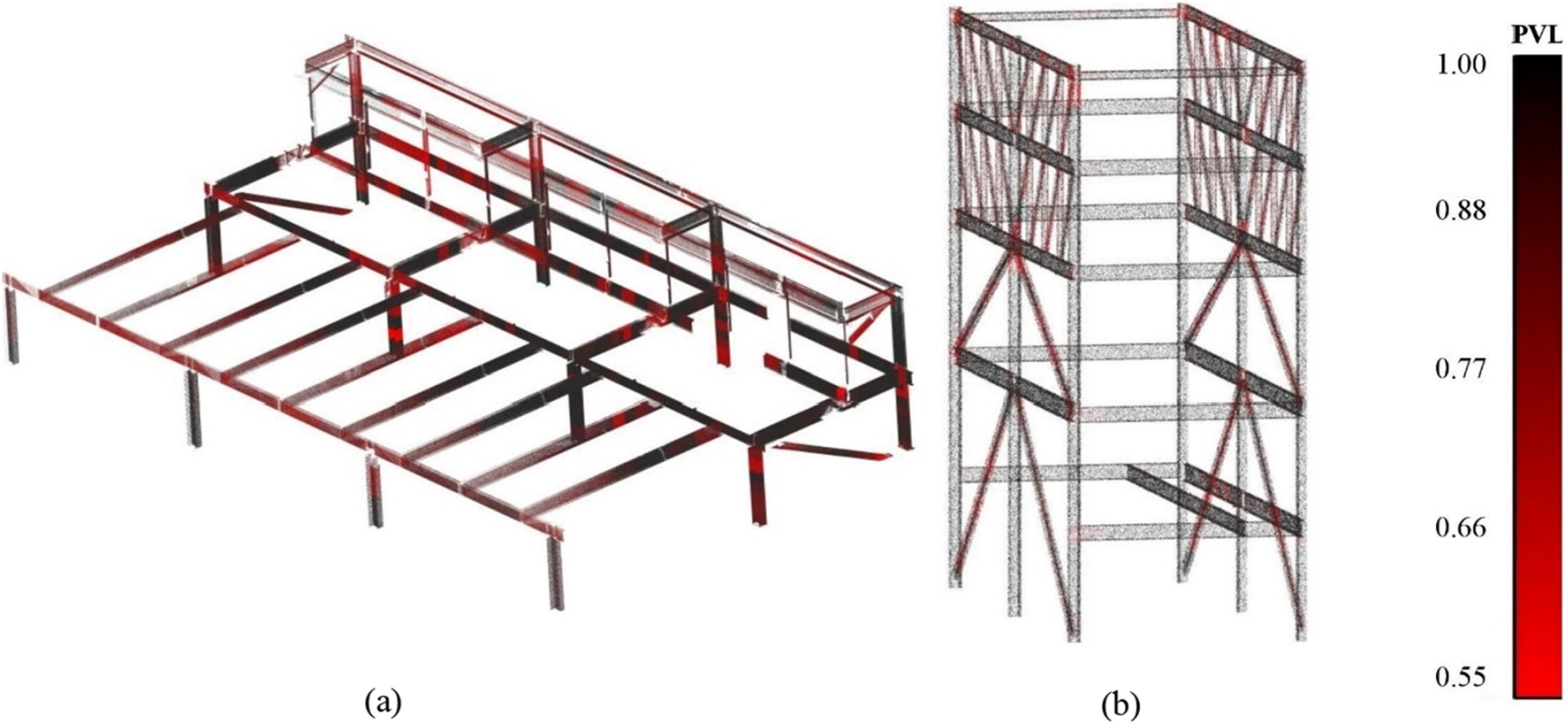
Probability vector length of the classified (**a**) multi-level carpark, and (**b**) Woodside building.

We utilized PointNet and trained it with a synthetically generated dataset and a slicing method for detecting section shape information. The trained network demonstrated satisfactory performance on the classification of the validation dataset with an average accuracy of 96% over different categories, as depicted in Fig. 4. The proposed method for identifying structural members represented a satisfactory performance of 94% accuracy if excluding “Not identified” sections from the results and 89% accuracy if considering “Not-identified” sections as incorrect predictions. The method mostly faced difficulty predicting cross-section shapes that were not fully captured during the scanning process. For example, the TLS device had a sharp angle for capturing structural members of the first floor in the multi-level carpark point cloud. Therefore, the point cloud quality was low in those areas, resulting in low prediction performance. Another challenge for the prediction model was detecting the bracing-beam connection members as they were not included in the training dataset, and the classification network correctly labeled them as “Not identified”. Also, the probability vector length of the correct prediction is mainly near the maximum value of one, demonstrating the model’s high confidence in predicting the correct section shapes, as shown in Fig. 5a,b for multi-level carpark and Woodside building point clouds, respectively.

Based on the results obtained from the section shape information step, the completeness of the point cloud significantly impacts the classification network’s performance. A shortcoming noted in the proposed method is that it relies on the similarity of training data and the case study point clouds in terms of noise level and point density. The impact of this phenomenon can be seen in the difference between the performance of detection of structural shapes in the Woodside building point cloud, which has a lower level of noise compared to the multi-level carpark case study as depicted in Fig. 5. Thus, it is suggested that future research focus on developing methods for generating training datasets with a dynamic level of noise and point density. In summary, the proposed method for obtaining geometric information from point clouds demonstrated satisfactory performance in getting position and section shape information, answering this paper’s first research question (Q1).

For inferring non-geometric information from point clouds, we applied a set of conditional rules based on the contextual knowledge definitions of building structural members. The function information was found based on the geometric information of the points. We fitted a bounding box around each member and utilized its dimensions for the type information. This method had a satisfactory performance in members without attached non-structural objects, such as MEP systems. The attached non-structural objects increased the bounding box’s size, resulting in incorrect size and type predictions in members. Thus, it is suggested that future research focuses on filtering out attached non-structural objects using RGB values before fitting the bounding box. Moreover, the homogenization step had a satisfactory performance for turning the obtained geometric information into construction-related information that can be used for various applications. The homogenization step considers the same weight for all section shape predictions. However, Fig. 5 shows that network confidence in predicting section shape differs in different point cloud slices. Therefore, future research must focus on developing a homogenization method that applies confidence weights to the section slices. We found that structural member function and section family type information can be inferred from point clouds, answering this paper’s second research question (Q2).

We used the Revit database to generate the BIM of structural members required for gDT models. The proposed method creates instances of objects in the Revit environment. One of the challenges observed is that object instances automatically created by the proposed API clash in the connection areas and require manual clash detection and removal procedures. Thus, one of the promising research areas for future endeavors is incorporating clash detection in the proposed API in the connection area of structural members. Also, connection types can be identified from the point cloud dataset to increase the level of details of the created BIM. While this paper focuses on generating a gDT of existing buildings through capturing the as-is condition of facilities in point clouds and processing that information offline to create a digital replica, a promising research area for future research is to create an online connection between the physical asset and the digital model with a feedback loop leading to the creation of DTs from gDTs. The online data received from the physical model can maximize the efficiency of DTs by providing a joint optimization decision-making system for the facilities^71^. Also, cyber-physical systems have demonstrated an excellent promise for integrating the virtual and physical worlds^14^. In summary, we found that our proposed method is not only capable of connecting geometric and non-geometric information to Revit’s dataset using the developed API for creating a BIM for various object types but also creates a platform for cyber-physical models to be implemented, integrating physical and virtual worlds, answering the third research question (Q3).

Lastly, we evaluated the performance of the proposed method for not only creating gDT’s virtual model from point clouds but also the service of health monitoring via comparing the final results obtained with the ground truth information, as shown in Table 3. Also, to evaluate the accuracy of the proposed method for creating BIM of building structural members to be used as gDT virtual models for structural health monitoring purposes, the structural members of the case study building were manually measured five times, and the average value was reported. The responsible measuring personnel reported an average tolerance of ± 25 mm, which is slightly higher than the values reported in previous literature^72,73^ due to difficulties in accessing building structure members for manual measurements.

**Table 3:** Comparison between the obtained dimensions and ground truth data for study building.

Case study building	Member mark	Length (mm)				Section shape detection accuracy		
		Ground truth	Obtained	Variation	Accuracy (%)	No. of correct section predictions	Total no. of structural members in the group	Accuracy (%)
Multi-level carpark	B1	8300	8267	33	99.60	9	9	100
	B2	6000	6012	12	99.80	3	4	75
	B3	25,760	25,750	10	99.96	1	1	100
	B4	25,760	25,769	9	99.97	1	1	100
	B5	25,760	25,780	20	99.92	1	1	100
	B6	25,760	25,733	27	99.90	1	1	100
	B7	2500	2512	12	99.52	3	4	75
	B8	25,760	25,736	24	99.91	1	1	100
	B9	25,760	25,779	19	99.93	1	1	100
	C1	2550	2523	27	98.94	3	4	75
	C2	2550	2536	14	99.45	4	4	100
	C3	5250	5263	13	99.75	3	4	75
	C4	2550	2522	28	98.90	7	10	70
	BR1	6200	6152	48	99.23	1	2	50
	BR2	3500	3435	65	98.14	1	2	50
		Average length accuracy: 99.52				Average section type detection accuracy: 81.63		
Woodside building	B1	9120	9102	18	99.80	7	8	87.5
	B2	23,320	23,305	15	99.94	7	9	77.77
	B3	9120	9142	22	99.76	2	4	50
	C1	24,450	24,440	10	99.96	6	6	100
	BR1	5000	4976	24	99.52	28	32	87.5
	BR2	3450	3429	21	99.39	21	24	87.5
	BR3	12,800	12,751	49	99.62	4	4	100
	BR4	10,200	10,239	39	99.62	3	4	75
		Average length accuracy: 99.7				Average section type detection accuracy: 86.31		

The proposed method demonstrated an error tolerance of ± 24.06 mm from ground truth in multi-level carpark and an error tolerance of ± 24.75 mm from ground truth in the Woodside building for obtaining the length of structural members from the automatically created BIM. Also, the model’s accuracy for detecting the length of structural members was calculated using Eq. (2).

2 $$Accuracy\, \left(\%\right)=100\times \left(1-\frac{\left|Obtained \, dimension-Ground \, truth \, dimension\right|}{Ground \, truth \, dimension}\right).$$

Finding the correct length of bracing members opposed a challenge for the proposed method as they show higher error tolerance compared to manual measurements, primarily due to the lack of data within the point cloud dataset. Thus, it is recommended that future research focuses on optimizing the scanning locations to fully capture the as-is state of construction and civil infrastructure projects. Also, the proposed method could correctly detect section family types and shapes with an average accuracy of 81.63% in multi-level parking and 86.31% in Woodside building for creating gDT’s virtual model. Also, the total processing time of the proposed method was 26 min on average for each case study building, which is generally shorter than traditional manual measurements. Overall, the proposed method promptly demonstrated a lower average error tolerance compared to manual measurement methods, showing its accuracy and efficiency to be utilized as a gDT creation method for structural health monitoring purposes, answering this paper’s fourth research question (Q4).

## Conclusion

An automatic method for creating BIMs as gDTs’ virtual models of building structural members (beams, columns, and bracings) was introduced in this work. The proposed method directly processed unorganized point clouds defined by the coordinates (x, y, z) as data input for gDT, obtained from terrestrial laser scanners, as connection input for DT. First, the method obtained geometric information such as position and section shape from the point cloud dataset using contextual hard-coded knowledge and geometric definition of structural members. The main geometric definitions used are: (1) points corresponding to beams are congested in horizontal slices within the point cloud, (2) points corresponding to columns have a horizontal normal vector, and (3) points associated with bracing members are located beneath beam points.

Then, PointNet, a deep neural network trained by a synthetic dataset, is used to detect the section shape of structural members. Next, structural sections were homogenized for each structural member, assuming that having multiple section shapes along a structural member in a regular building is rare. After obtaining the geometric information, contextual knowledge and conditional statements were used to infer non-geometric information. Non-geometric information for creating a BIM consists of structural members’ function and family type information. Lastly, geometric and non-geometric information was connected to the database of Revit by developing an API for creating a BIM as gDT’s virtual model.

The results indicated an error tolerance of ± 24.06 mm from ground truth in multi-level carpark and error tolerance of ± 24.75 mm from ground truth in Woodside building for obtaining the length of structural members from the automatically created gDT virtual model. Also, the proposed method could correctly detect section family types and shapes with an average accuracy of 81.63% in multi-level parking and 86.31% in Woodside building for creating the gDTs’ virtual models. Overall, it is concluded that the proposed automatic method for gDT generation using raw point cloud data stands out in terms of accuracy and efficiency compared to both traditional manual methods and previously proposed approaches.

Future work includes developing methods for reducing the time required for obtaining and registering as-is point clouds of construction and infrastructure projects. Also, more categories of construction-related objects, such as structural members’ connections, will be added to the method to enhance the proposed approach’s generality. Finally, a promising research area for future research is to create an online connection between the physical asset and the digital replica with a feedback loop to integrate virtual and physical spaces using the platform developed in this study.

## Materials and methods

**Figure 6:**
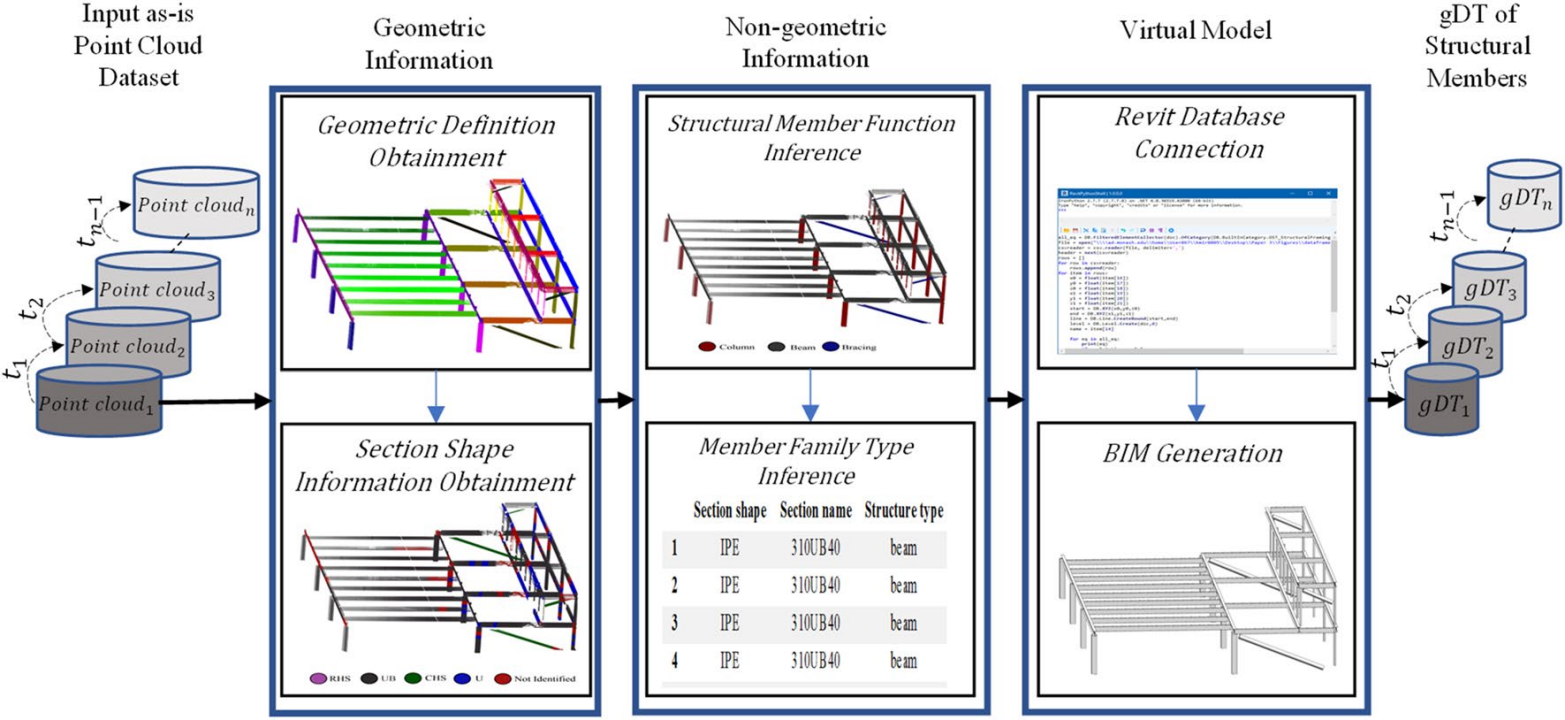
The workflow for the geometric digital twin-driven dimensional quality inspection of building structural members.

The BIM approach for the gDT generation of structural members in construction and civil infrastructure projects from point clouds is summarized into four significant steps: 1. Point cloud acquisition and preprocessing 2. Geometric information obtainment 3. Non-geometric information inference, and 4. BIM generation for creating/updating the gDT model. The proposed method workflow is visually illustrated in Fig. 6.

### Point cloud acquisition and preprocessing

Point cloud data were obtained by a terrestrial laser scanner (FARO® Focus M70) from the interior and exterior environment of (a) multi-level carpark, and (b) Woodside building to validate the performance of the proposed gDT generation method, as shown in Fig. 1. A total of 11 scans from the interior of each case study building with ¼ resolution and 4× quality settings were obtained with the average point number of $3\times {10}^{6}$ points in each scan. The same scan parameters were used for both case study buildings to demonstrate the impact of different noise and point densities on the overall performance of the proposed method. Then, a point cloud registration was performed to merge the obtained scans from the case study building into a complete 3D point cloud using FARO® SCENE Software. The registration took 10–12 h using a desktop computer (Intel i7-9700 CPU @ 3.00 GHz, 32 GB RAM, and 500 GB SSD), resulting in a complete 3D point cloud for each building. Finally, a mixed part of the point cloud from buildings was segmented manually to evaluate the proposed method’s performance and reduce computational time. Specifications of the laser surveys for obtaining point clouds of case study buildings are given in Table 4.

**Table 4:** Metadata of the case study buildings point cloud datasets.

Metadata	Multi-level carpark	Woodside building
No. of scans	11	11
No. of points in the point cloud	3,342,352	2,281,412
Scanner model	FARO® Focus M70	FARO® Focus M70
Scanner range	0.6–70 m	0.6–70 m
Ranging errors at 10 m and 25 m	± 1 mm	± 1 mm
Registration error	± 3 mm	± 4 mm

The role of a gDT is to be a dynamic digital representation of an asset during different life cycle phases. Thus, a gDT must be updated frequently due to unavoidable changes in the as-is condition of an asset over time (i.e., gDT_1_, gDT_2_, gDT_3_,…, gDT_n_). The frequency (i.e., t_1_, t_2_, t_3_,…, t_n−1_) of capturing as-is data (i.e., point cloud_1_, point cloud_2_, point cloud_3_,…, point cloud_n_) and updating a gDT model is related to the service component of gDT. For structural health monitoring of buildings, an optimized frequency of capturing as-is data leads to in-time detection and rectifying defects with the right level of resources and inspection costs. Since structural health monitoring of buildings is a soft real-time task, in which the deadlines can be allowed for delays as long as the tasks are timely executed, a risk-based approach should be used to find the optimal frequency of capturing as-is data to update gDT. While the minimum frequency of capturing as-is data for structural health monitoring purposes is stated in standards and regulations for different construction projects^74^, an increase in the level of building importance, defect risk, and hazard consequences can further escalate the frequency of capturing as-is data. Therefore, in consultation with designers, the statutory building inspector can set the frequency of capturing as-is data for a soft real-time structural health monitoring of buildings using the framework proposed in Fig. 6.

The preprocessing steps are primarily designed to (1) reduce the computational burden of point cloud processing steps by removing floor and ceiling points within the dataset and (2) align structural members with the point cloud coordinate system. For the first step, Ceiling and floor planes are filtered out by an improved RANSAC algorithm operating on Normal Distribution Transformation (NDT) cells^75^ through classifying point clouds into planar and non-planar cells. For aligning the structural members with the point cloud coordinate system, points within the point cloud should be multiplied by a transformation matrix as a vector (x, y, z), as shown in Eq. (3).

3 $${R}_{z}\left(\theta \right)=\left[\begin{array}{ccc}\mathrm{cos}\left(\theta \right)& -\mathrm{sin}\theta & 0\\ \mathrm{sin}\theta & \mathrm{cos}\left(\theta \right)& 0\\ 0& 0& 1\end{array}\right],$$

where $\theta$ is the rotation angle about the Z-axis since the Z-axis is correctly adjusted during the scanning and registration processes. While angle $\theta$ can be determined manually using visualization software such as Cloud Compare, Principal Component Analysis (PCA) method is used for automated and exact identification of angle θ^76^.

### Geometric information obtainment

**Figure 7:**
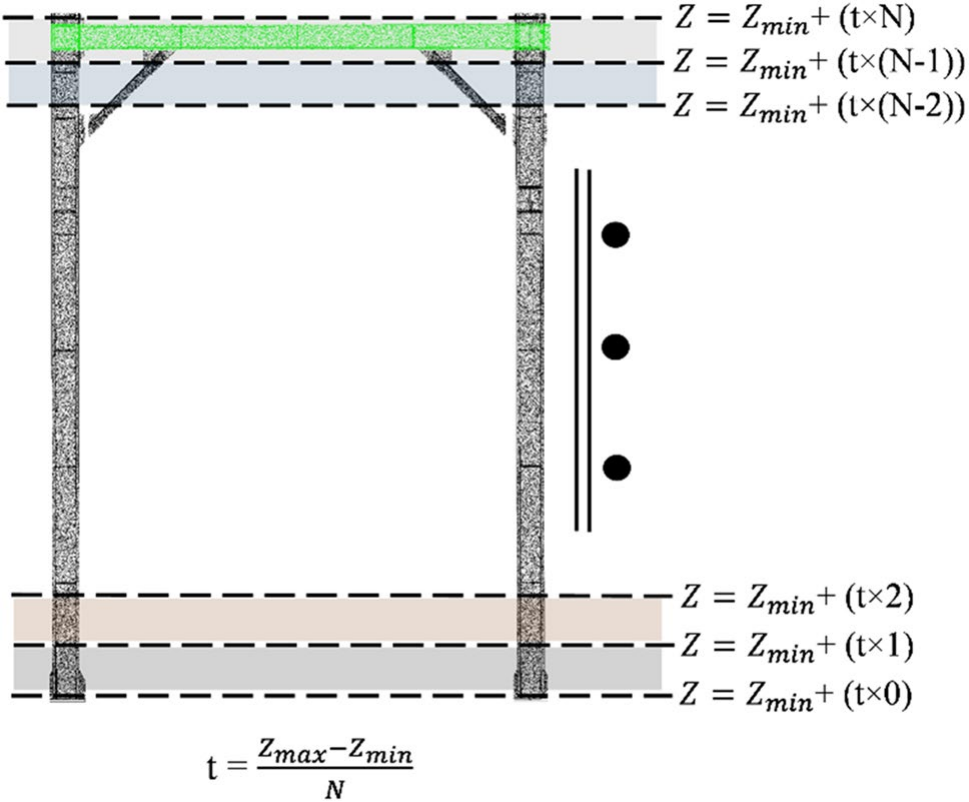
Horizontal slicing of a frame.

Generating structural members’ BIM as gDT’s virtual model requires geometric and non-geometric information from point clouds. The first group of geometric information is position information. In the position information step, points possessing geometric definitions and relationships of building structural members are segmented. Building beams are defined as horizontal members parallel to support horizontal structures like floors. Thus, it can be geometrically inferred that beam points are congested in horizontal slices within the point cloud dataset. Therefore, a slicing method is applied to create horizontal point cloud slices, as shown in Fig. 7. An optimal value of the horizontal slices can be found by using contextual information of standard structural section dimensions (e.g., AS/NZS 3679.1:2016^65^) as shown in Eq. (4) as

4 $$\frac{{Z}_{\mathrm{max}-}{Z}_{min}}{N}\le \frac{{d}_{s}}{2},$$

where ${Z}_{max}$ and ${Z}_{min}$ are the maximum and minimum Z-axis coordinates, respectively, ${d}_{s}$ is the minimum difference between the height of standard structural sections as stated in standards, and $N$ is the number of horizontal slices. After finding the $N$, the thickness of horizontal slices is calculated using Eq. (5) as

5 $$t=\frac{{Z}_{\mathrm{max}-}{Z}_{min}}{N},$$

where $t$ is the thickness of the horizontal slices. Next, points are distributed between slices $i$ and $i+1$ using their Z-axis coordinates as Eq. (6) as

6 $${Z}_{min}+\left(t\times i\right)\le z\le {Z}_{min}+\left(t\times \left(i+1\right)\right),$$

where $0\le i\le N$. After distributing the points between horizontal slices, the number of points within each horizontal slice is statistically compared to find the slices with a relatively higher density of points. The outcome of this step is beam points associated with each floor of the building. Since point cloud segmentation aims to detect different instances of structural members, the MeanShift clustering algorithm is used to segment the beam points of each floor based on the X and Y coordinates into single instances of beam members. Next, columns will be geometrically detected within the point cloud dataset. Columns are geometrically defined as vertical structural members for transferring compressive force in the building. Points belonging to vertical members within a point cloud dataset can be detected by calculating the normal vector of that point as an array of [${n}_{x},{n}_{y}, {n}_{z}$], in which if the absolute value of ${n}_{z}$ is close to zero, that point can be considered a potential column point. The outcome of this step is a group of points that have the geometric definition of a column; therefore, the MeanShift algorithm is used to cluster out different instances of columns based on the X and Y coordinates of points. Lastly, bracings are geometrically defined as diagonal members existing beneath beams in buildings. Thus, points associated with bracings in a point cloud dataset can be geometrically detected as points with the X and Y coordinates within each beam’s X and Y boundaries with a Z coordinate less than the minimum Z coordinate of beam points.

The next group of geometric information required for generating gDT of building structural models from a point cloud dataset is section shapes of the geometrically segmented members obtained from the previous step. A supervised classification method is proposed for detecting different section shapes of geometrically segmented point clouds. For this purpose, PointNet, a deep neural network, is utilized for predicting the section shape of each geometrically segmented point cloud^53^. PointNet uses a symmetric function (i.e., max pooling) that is order-invariant to act as a local feature learner and aggregate local features to acquire global features from each point. Thus, PointNet architecture can deploy multilayer perceptrons without converting point clouds into structured grid datasets for classifying the shape of geometrically segmented members.

PointNet heavily relies on precise and abundant training data as a deep learning method. Thus, a synthetic training dataset of structural shapes is proposed to compensate for the lack of a publicly available dataset of structural section shapes. This dataset consists of point cloud sections automatically generated from 3D shapes. Also, random levels of noises, rotations, and occlusions are applied to the training dataset to increase its similarity to real-world point clouds. While PointNet trained by synthetic dataset is capable of being directly used on geometrically segmented members for classifying point cloud section shapes, the length of geometrically segmented point clouds opposes a problem for the classification network as the network considers the members’ length as the main feature of the point cloud instead of its section shape. Thus, prior to applying PointNet for classifying section shapes, geometrically segmented members are sliced into smaller sections to increase the classification network’s performance.

The output of the classification network on each point cloud slice is a probability distribution over the set of section categories. Therefore, the confidence of the classification network is inferred by calculating the PVL of each classified point cloud slice using Eq. (1). In Eq. (1), n is the size of the probability vector population, and σ is the standard deviation of the probability vector population. The value of PVL highlights the differences between the probability values in the classification network outcome’s probability distribution. A higher value of PVL means a more significant disparity between probability values, showing a higher confidence level for the classification network. Finally, the section label will be added to each point cloud slice as an attribute required for generating a BIM model. Section shapes identified with low confidence are labeled as “Not identified”, which the user should manually label.

### Non-geometric information inference

Non-geometric information, such as member function and section family type, is necessary for generating the BIM as a gDT virtual model of structural members. However, only geometric information can be directly obtained from point clouds. To cover this gap, contextual knowledge of structural members is used for inferring non-geometric information from geometric information. Member function information is the first non-geometric information required to create a structural members’ gDT virtual model. First, the section shape along each structural member should be homogenized since structural members were sliced in the semantic segmentation step, and each can have a different section label. To homogenize section shapes along a structural member, the section shape with the most significant number of points is assigned as the section shape for the whole structural member. A conditional rule is defined for inferring member function from geometric information as “if a point is geometrically labeled as a structural member AND geometrically labeled to be a part of structural section shape, then it can be inferred that the member function of that point is the same as its geometrical definition label”. This conditional rule filters out non-structural objects possessing structural members’ geometric definitions from the point cloud.

The next group of non-geometric information required for generating BIM as gDT virtual replica of structural members is section family type. This information is inferred by drawing a bounding box around each member to get section size (i.e., section width, height, length) and conditional rules to detect the subcategory of section shapes (i.e., IPE and IPB in the UB category). For labeling the section dimensions, the maximum and minimum values of X, Y, and Z coordinates are subtracted from each other. Their highest value corresponds to the elongation of the structural member and is labeled as member length. As for the other two values, the smaller one is labeled as section width, and the other is labeled as section height. For members with the UB section label, if the height is 50% bigger than the width, that section is labeled as IPE; otherwise, it would be labeled as IPB. To find the start and end point of the section, the coordinate of the center of mass of the section is calculated, which is the same in both starting and endpoints. For the third coordinate, the maximum value of the elongation dimension is the endpoint, and the minimum value is the starting point. Next, the obtained information from each structural member is saved within a database.

### BIM generation

Finally, Revit generates the BIM model as gDT’s virtual model. The underlying architecture of Revit is a database used to store and share information. Thus, after obtaining the necessary information for generating a BIM model, the obtained database is connected to the Revit database to automate the generation of BIM models. To reach this purpose, first, a list of all of the family symbols loaded in the Revit is acquired by applying the “FilteredElementCollector” command within the Revit dataset. Then, for each structural member, if the obtained section name from the point cloud matches any section names in the Revit database, that section name in the Revit database and its corresponding family symbol would be chosen as the input for the “CreateNewFamilyInstance” command in Revit. Next, the member starting and endpoints saved in the database obtained from labeled point clouds are used for defining the line on which the structural member is drawn in Revit. Finally, the structural function (i.e., beam, column, bracing) read from the point cloud dataset is the final input for creating the new family instance inside Revit. After repeating the procedure for all structural member point clouds, the BIM required for gDT is generated. Table 5 includes the pseudocode for creating BIM from the geometric and non-geometric information obtained from case study buildings’ point clouds.

**Table 5:** Pseudocode for automatically creating BIM using information obtained from point clouds.

**Input** The output of “Geometric information obtainment” and “Non-geometric information inference” steps stored in a dataset containing each member’s boundary coordination, section shape, section family name, and structural function **Output** BIM in Revit environment as gDT’s virtual model
**BIM generation process** 1. Select the active document in Revit (doc) 2. Select all of StructuralFraming objects loaded in doc using FilteredElementCollector (all_eq) 3. Load the database file into API (file) 4. Read the database file (csvreader) 5. For row in csvreader do 6. Select the start coordination stored in the database (start) 7. Select the end coordination stored in the database (end) 8. Draw a line between the start and end points (line) 9. Select the floor number stored in the database (level) 10. Extract section family name from the database (name) 11. Extract structure member function from the database (function) 12. For equipment in all_eq do 13. If name==equipemnt’s name do 14. Create NewFamilyInstance using line, name, level, function 15. End if 16. End for 17. End for

## Data Availability

All data generated or analyzed during this study are included in this published article.
